# The impact of peer relationships and employment pressure on the learning wellbeing of university students in the post-2000s generation

**DOI:** 10.3389/fpsyg.2025.1688537

**Published:** 2026-01-09

**Authors:** Huajie Shen, Xinzhi Ye, Caixia Bai, Shi He, Fengwu Zhang, Yushan Yang, Jian Qiu

**Affiliations:** 1 School of Education, Fujian Normal University, Fuzhou, Fujian, China; 2 School of Design, Fujian University of Technology, Fuzhou, Fujian, China; 3 College of Material and Chemical Engineering, Southwest Forestry University, Kunming, Yunnan, China

**Keywords:** peer relationships, employment pressure, learning wellbeing, post-2000s university students, interaction effects

## Abstract

Learning wellbeing has become an important indicator of university students’ academic experience and psychological adjustment. Guided by a correlational and non-causal analytical framework, this study examined how interpersonal distress (as an indicator of low peer relationship quality) and employment pressure are related to the learning wellbeing of post-2000s university students in China. A total of 600 undergraduates from four universities participated in the survey. Standardized questionnaires and quantitative analysis methods were used to assess interpersonal distress, employment pressure, and four dimensions of learning wellbeing (cognitive, emotional, quality-of-life, and growth wellbeing). Quantitative analysis methods, including, descriptive statistics, Pearson correlations, and multiple regression analyses were applied to examine the relationships and interaction effects between these variables. The results showed that interpersonal distress was significantly and negatively associated with all dimensions of learning wellbeing. Employment pressure demonstrated differentiated associations: moderate pressure was positively related to cognitive and emotional wellbeing, while no significant direct association was found with quality-of-life or growth wellbeing. Interpersonal distress also played a modest moderating role: higher interpersonal distress was associated with less favorable patterns of learning wellbeing under employment pressure, whereas lower interpersonal distress was associated with relatively more favorable patterns. However, the interaction effects explained only a small proportion of variance, and the findings should therefore be interpreted with caution. Overall, the study suggests that interpersonal distress and employment pressure are connected with university students’ learning wellbeing in complex and multidimensional ways. The findings align with and extend existing literature by highlighting the relational context in which students experience academic and employment-related demands. Implications for campus mental health services and career counseling, as well as directions for future longitudinal research, are discussed.

## 1 Introduction

With the ongoing evolution of higher education structures and pedagogical philosophies, university students’ learning wellbeing has become a central topic in educational psychology and higher education administration. Learning wellbeing not only concerns students’ subjective experiences and positive affect in academic activities but is also directly associated with their academic achievement, self-development, and overall psychological adjustment (Liu et al., 2024).

In today’s increasingly competitive university environment, the factors influencing students’ learning wellbeing have diversified. Among these, peer relationships and employment pressure have received growing scholarly attention as two highly salient variables. On the one hand, peer relationships function as a crucial social support system, providing important resources for learning, daily life, and emotion regulation. High-quality peer interactions help strengthen students’ sense of belonging, academic confidence, and interpersonal adaptation (Cao et al., 2024). On the other hand, employment pressure constitutes a key psychological burden during career preparation; its intensity and frequency often affect students’ academic motivation, goal commitment, and psychological resilience, thereby shaping the formation and maintenance of learning wellbeing (Capone et al., 2021).

Against this backdrop, drawing on empirical data, this paper systematically examines the pathways through which peer relationships and employment pressure affect the learning wellbeing of Post-2000s generation university students. It investigates both their direct effects and interactive mechanisms, with the aim of providing a theoretical basis and practical implications for universities’ psychological support and career counseling.

With the development of positive psychology, learning wellbeing has gradually become an important topic in the field of higher education. Existing studies have shown that learning wellbeing is closely related to university students’ academic engagement, academic achievement, and persistence in learning, and is also significantly negatively associated with psychological problems such as depression and anxiety. It is therefore regarded as a key psychological indicator of the quality of students’ learning experiences and their developmental status (Mahmoodi et al., 2019; Tian et al., 2021). However, the current literature still has several notable limitations.

First, many studies treat wellbeing as a global life-domain construct and focus primarily on life satisfaction or general subjective wellbeing. Relatively few studies have taken learning wellbeing itself as the core outcome variable, and the internal structure of wellbeing in learning contexts and its specific antecedent factors have not been examined in sufficient depth. Second, research on influencing factors typically focuses on either peer relationships or employment pressure. One line of work emphasizes the positive role of social support and peer interactions in students’ subjective wellbeing and school adjustment, whereas another line highlights the negative impact of employment conditions and career prospects on university students’ stress levels and mental health. Only a small number of studies have incorporated peer relationships and employment pressure into a single analytical framework to systematically investigate their associations with learning wellbeing and their potential joint effects. Third, for the Chinese “post-2000″ generation of university students, who are facing increasingly intense employment competition and complex, changing campus interpersonal environments, empirical research remains limited, especially with respect to evidence on the interaction between interpersonal distress (low-quality peer relationships) and employment pressure in relation to learning wellbeing.

In light of these gaps, the present study focuses on Chinese “post-2000” university students and, from the perspective of the learning context, examines the associations between peer relationship quality (indexed by interpersonal distress), employment pressure, and learning wellbeing. Moreover, we further analyze whether the interaction between peer relationship quality and employment pressure is related to different levels of learning wellbeing. By simultaneously incorporating interpersonal and employment-related stress, this study aims to: (1) deepen the understanding of multiple determinants of learning wellbeing and complement existing research that has mainly focused on problem-oriented outcomes; (2) reveal the joint patterns of interpersonal distress and employment pressure in relation to learning wellbeing, thereby providing a basis for targeted support and intervention in universities; and (3) offer context-specific empirical evidence on the subjective wellbeing of Chinese “post-2000” university students during the transition from study to employment.

The structure of this study is arranged as follows. Section 2 reviews relevant theories and empirical studies, defines the core constructs of learning wellbeing, peer relationships (interpersonal distress), and employment pressure, and presents the research hypotheses. Section 3 describes the research design, including the sample and sampling procedures, measurement instruments, and data analysis methods. Section 4 reports the results of the descriptive statistics, correlation analyses, and regression and interaction analyses. Section 5 discusses the main findings in light of existing research, elaborates on their theoretical and practical implications, points out the limitations of the study, and proposes directions for future research.

## 2 Literature review

### 2.1 Learning wellbeing: theoretical foundations, conceptual definition, and measurement

#### 2.1.1 Conceptual definition and connotation of learning wellbeing

Learning wellbeing is generally regarded as a specific manifestation of subjective wellbeing in educational and learning contexts. It refers to students’ overall positive evaluation of their learning life over a given period of time, as well as the relatively enduring and stable positive emotional states (e.g., enjoyment, engagement, satisfaction) they experience during learning, accompanied by relatively low levels of negative emotions (e.g., anxiety, frustration) (Zuhdiyah et al., 2020).

Similar to subjective wellbeing in general life domains, learning wellbeing comprises both a cognitive evaluation component and an affective experience component. The former is mainly reflected in students’ satisfaction with their learning outcomes, learning processes, and overall quality of learning life, whereas the latter is reflected in the frequency and intensity of positive emotions experienced during learning, along with the relative infrequency of negative emotions (Kyte, 2016).

In the context of higher education, learning wellbeing also exhibits salient developmental and value-oriented characteristics. University students are concerned not only with grades and competence enhancement, but also with whether their learning experiences contribute to self-realization, clarification of life goals, and the fulfillment of personal potentials (Wu, 2024). Accordingly, in this study, learning wellbeing among university students is conceptually defined as:a comprehensive psychological state in which students, within the university learning context, hold an overall positive cognitive evaluation of their learning-related experiences and meanings, and continuously experience positive emotions, a sense of growth, and a sense of value in the process of learning.

#### 2.1.2 Theoretical foundations of learning wellbeing

Psychological research on wellbeing has primarily developed along two major theoretical traditions: subjective wellbeing (SWB) and psychological wellbeing (PWB). Together, these traditions provide an important foundation for the conceptualization and measurement of learning wellbeing.

Diener (1984) proposed that subjective wellbeing consists of three relatively independent but interrelated components: life satisfaction (cognitive evaluation), the frequency of positive emotional experiences, and the relative infrequency of negative emotional experiences. He emphasized that wellbeing represents a combination of individuals’ subjective judgments and emotional reactions regarding their life quality. Subsequent research has further shown that this three-factor model not only applies to general life domains but can also be transferred to educational settings to describe students’ affective experiences and satisfaction with school life and learning activities (Qin et al., 2025).

In learning contexts, based on the theory of subjective wellbeing, the core of learning wellbeing lies in the following: students experience more positive emotions such as interest, enjoyment, concentration, and a sense of achievement during learning, experience fewer negative emotions such as anxiety, helplessness, and boredom, and hold a high level of satisfaction with their academic performance, learning environment, and overall learning life (Dong et al., 2025).

In contrast to subjective wellbeing, which emphasizes emotions and satisfaction, the theory of psychological wellbeing focuses more on the optimization of personality development, self-realization, and social functioning. Integrating multiple theoretical perspectives from developmental and humanistic psychology, Ryff (1995) proposed that psychological wellbeing can be divided into six dimensions: self-acceptance, positive relations with others, autonomy, environmental mastery, purpose in life, and personal growth, and developed a relatively systematic set of measurement tools accordingly.

Building on this work, Ryan and Deci (2001), drawing on self-determination theory, argued that the attainment of wellbeing fundamentally depends on the sustained satisfaction of three basic psychological needs: autonomy, competence, and relatedness. When individuals perceive their behavior in learning and life as self-determined, experience competence enhancement, and maintain positive connections with others, their wellbeing tends to be higher and more stable.

Within the context of university learning, the psychological wellbeing perspective highlights the developmental and meaning-oriented connotations of learning wellbeing: students feel that their abilities are continuously improving through learning (competence), that they can exercise a certain degree of choice and regulation over the learning process (autonomy), and that they establish positive interpersonal bonds (relatedness) through interactions with peers and teachers, while holding a clear value-based commitment to learning goals and their linkage to future development (Morales-Rodríguez et al., 2020).

Taken together, these two theoretical traditions suggest that learning wellbeing can be examined at both the emotional–cognitive level (e.g., learning satisfaction, positive and negative academic emotions) and the developmental–meaning level (e.g., perceived growth, sense of purpose, and self-realization in learning). Accordingly, at the theoretical level, the present study conceptualizes university students’ learning wellbeing as a multidimensional construct consisting of cognitive evaluation (learning satisfaction), affective experience (positive and negative learning emotions), and developmental meaning (sense of growth and value). These components correspond to the core dimensions of subjective and psychological wellbeing and provide the basis for our conceptualization and measurement of learning wellbeing.

#### 2.1.3 Measurement of learning wellbeing

At the measurement level, research on wellbeing generally adopts self-report questionnaires and uses Likert-type scales to collect individuals’ subjective reports of their wellbeing states. Overall, there are two main approaches to measuring learning wellbeing: (1) contextualizing general subjective wellbeing scales for use in learning settings, and (2) developing school/learning domain-specific wellbeing scales.

In general life domains, subjective wellbeing is commonly measured using indicators such as life satisfaction and positive and negative affect (Diener, 1984). Some studies have adopted the same measurement approach in school settings by explicitly framing item statements within “at school/in learning” contexts, thereby obtaining domain-specific indicators of students’ subjective wellbeing in learning. For example, based on Diener’s three-factor model, Long et al. (2012) proposed the structure of school-related subjective wellbeing and, using confirmatory factor analysis with a sample of 921 adolescents, confirmed that a four-factor model consisting of school satisfaction, positive affect in school, negative affect, and fear-related affect in school showed good fit in adolescent samples (Long et al., 2012). However, generalized wellbeing scales may not fully capture the unique goal structures, interpersonal environments, and developmental tasks present in learning contexts, which has led to the development of more context-specific instruments.

In the Chinese context, Tian and colleagues have developed several scales for assessing subjective wellbeing in school settings, including the Adolescents’ Subjective Well-Being in School Scale and the Brief Adolescents’ Subjective Well-Being in School Scale. Multiple studies have provided evidence for their satisfactory reliability and structural validity (Tian et al., 2014, 2016). These scales typically conceptualize school-related wellbeing as a combination of students’ subjective evaluations of their school life and the positive and negative emotions they experience at school.

In international research, Renshaw et al. (2015) developed the Student Subjective Wellbeing Questionnaire (SSWQ), which assesses students’ school-related wellbeing across four dimensions: joy of learning, school connectedness, educational purpose, and academic efficacy. This instrument highlights the importance of perceived meaning in learning and academic efficacy in the structure of students’ wellbeing (Renshaw et al., 2015).

Drawing on the above theoretical and measurement advances, the present study employs the College Students’ Learning Well-Being Questionnaire developed by Gao (2014) to assess learning wellbeing among university students. This questionnaire is grounded in both subjective wellbeing and psychological wellbeing theories and includes two subscales: subjective learning wellbeing and psychological learning wellbeing. It covers four dimensions—cognitive wellbeing, emotional wellbeing, quality wellbeing, and growth wellbeing. Conceptually, this dimensional structure is highly consistent with the dual-component (emotional–cognitive) model of subjective wellbeing (Diener, 1984) and with the psychological wellbeing dimensions related to personal growth, purpose in life, and positive relations with others (Ryff, 1995; Ryan and Deci, 2001). Therefore, from a theoretical perspective, the learning wellbeing scale adopted in this study can comprehensively capture university students’ emotional experiences, subjective satisfaction, and developmental meaning in the learning domain. It is consistent with classic theories of subjective and psychological wellbeing and also meets the needs of localized research on learning wellbeing in the Chinese context.

### 2.2 Definition and measurement of peer support

#### 2.2.1 Conceptual definition of peer support and interpersonal distress

“Peer support” is a key component of the social support system and has long been a subject of interest across various disciplines, including psychology, medicine, and education. However, a unified definition has yet to be established. Early research generally viewed “peers” as individuals who share certain similarities in terms of age, status, abilities, and cognitive level (Foot and Morgan, 1990). In educational psychology, Wentzel et al. (2010) identified peer support as emotional care and practical assistance provided in academic and behavioral development. Bao (2025) confirmed in the field of sports psychology that peer relationships enhance exercise self-efficacy through dual pathways of emotional support and behavioral engagement. Utvær et al. (2022), from the perspective of crisis coping, revealed the instrumental value of peer support in digital learning, emphasizing its role in alleviating emotional stress and maintaining professional competence perception. Together, these studies highlight the integrative role of peer relationships in emotional connection and practical problem-solving.

In the context of higher education, peer relationships constitute one of the most important social resources for university students. Existing research generally regards peer support as a key component of the broader social support system, typically encompassing emotional support (e.g., understanding, care), informational/academic support (e.g., sharing learning experiences, academic guidance), and instrumental support (e.g., providing resources, helping to solve problems) (Worley et al., 2023; Fadiji and Eloff, 2024).

A large body of empirical evidence has shown that peer support is positively associated with students’ academic self-efficacy, school belonging, subjective wellbeing, and mental health. For example, peer support has been found to have prospective associations with increased academic competence and reduced anxiety among university students (Worley et al., 2023). Among children and adolescents, peer acceptance and the number of friendships are positively related to life satisfaction and academic achievement (Țepordei et al., 2023). In school settings, peer mutual assistance and positive learning communities help enhance students’ engagement and learning experiences (Brierley et al., 2022).

However, students may not only receive positive support in peer relationships; they may also experience negative interactions such as interpersonal conflict, rejection, and isolation. In recent years, increasing attention has been paid to negative interpersonal experiences such as interpersonal stress and interpersonal distress and their impact on university students’ psychological functioning and academic adjustment (Kao and Cheng, 2023). Studies with Chinese university students indicate that interpersonal stress is highly prevalent during this developmental stage and constitutes an important source of stress. Higher levels of interpersonal stress are often associated with greater psychological distress, lower subjective wellbeing, and poorer academic adjustment (Wang et al., 2023; Xu et al., 2023).

Theoretically, peer support and interpersonal distress can be viewed as two poles of the same underlying relational dimension. At one pole are stable, reciprocal, and supportive peer relationships that provide emotional warmth, a sense of security, and academic assistance. At the other pole are frequent interpersonal conflicts, communication barriers, and experiences of rejection, which generate substantial psychological pressure and feelings of isolation. The former tends to promote students’ learning wellbeing, school belonging, and subjective wellbeing, whereas the latter is linked to risk outcomes such as depression, anxiety, and academic burnout.

Within the specific context of learning wellbeing, the present study conceptualizes the peer relationship variable as the combined characteristics of support and distress that university students experience in their interactions with same-age peers. Positive peer support refers to emotional, informational, and instrumental help provided by peers in learning and everyday life, whereas interpersonal distress refers to recurrent negative experiences in peer interactions, such as tension, conflict, communication difficulties, and rejection. Given that the scale used in this study primarily assesses interpersonal distress, our subsequent analyses focus on the role of low peer relationship quality / high interpersonal distress.

#### 2.2.2 Measurement of peer support

Several multidimensional and context-specific localized questionnaires have been developed to measure peer support. Li et al. (2010), based on international questionnaire frameworks, constructed a social support scale that includes dimensions such as school environment, teacher-student interaction, and peer relationships, demonstrating good reliability and validity. Wang et al. (2018) refined the measurement indicators into four functional dimensions: informational advice, emotional encouragement, behavioral modeling, and companionship supervision, making it more applicable to the daily interactions and sports participation of adolescents. Mostafaei Alaei and Hosseinnezhad (2020) developed the Peer Support Questionnaire (PSQ), which includes five dimensions: informational, emotional, instrumental, validation/feedback, and companionship support. Factor analysis confirmed its structural validity, and it demonstrated good reliability, making it an effective tool for assessing students’ perceptions of peer support.

In conclusion, the current measurement tools have evolved from structural-functional to multidimensional, from singular environments to diverse contexts, effectively quantifying peer support in both cognitive and behavioral dimensions. These tools provide a reliable foundation for measurement in this study.

### 2.3 Employment pressure: theoretical foundations, conceptual definition, measurement, and intervention

#### 2.3.1 Theoretical foundations of stress

The concept of “stress” was first introduced by physiologist Selye (1978), referring to the psychological and physiological tension experienced by individuals when faced with external demands that exceed their coping resources. In psychology, three main theoretical perspectives have developed to explain stress mechanisms: the stress response theory, the social stimulus theory, and the cognitive transactional theory.

The Response-based theory, originating from physiology, posits that when individuals are exposed to external stimuli, they generate activation responses through their physiological systems (e.g., changes in heart rate, gastrointestinal disturbances), and the intensity of these physiological responses can serve as an indicator of stress levels (Chang and DeSimone, 2001). The Stimulus-based theory, proposed by Holmes and Rahe (1967), emphasizes that any life event, whether positive or negative, that alters an individual’s original life patterns, may induce psychological stress and health risks. The Transactional theory, integrating perspectives from sociology and cultural psychology, argues that stress does not arise from the situation itself, but from the individual’s subjective evaluation of the environment. Stress emerges when the situation is perceived as threatening to an individual’s needs or goal achievement (Sapolsky, 1987).

Among the many classic theories of stress, the one most directly relevant to employment pressure among university students is the transactional model of stress. This model emphasizes that stress does not simply arise from external stimuli or physiological reactions; rather, it is the subjective outcome of individuals’ appraisal of external demands (e.g., employment competition, economic conditions, social expectations) in relation to their own resources (e.g., abilities, self-efficacy, social support) (Lazarus and Folkman, 1984; Chang and DeSimone, 2001). When individuals appraise a situation as a threat that “exceeds their resources or undermines important goals,” they are more likely to experience persistent psychological stress and negative emotional reactions.

Among university students, the employment context is characterized by high uncertainty, intense competition, and strong social expectations, and is therefore often regarded as a prototypical high-stress situation. Recent studies have shown that employment pressure among university students is significantly associated with mental health indicators such as depression and anxiety, and has negative implications for their subjective and psychological wellbeing (Wu and Lin, 2025; Mofatteh, 2020).

Taken together, from the perspective of the stress–coping framework, employment pressure among university students can be understood as students’ subjective, threat-related appraisal of “uncertain employment prospects, highly competitive contexts, and multiple social expectations” during the transition to the labor market, as well as the resulting experiences of sustained tension, worry, and burden. This process is closely related not only to students’ mental health, but also to their learning motivation and level of learning wellbeing during their time at university.

#### 2.3.2 Conceptual definition of employment pressure

“Employment Pressure” is a specific application of stress theory in the career preparation phase of university students. Although there is no unified definition, most research perspectives have reached a consensus: employment pressure refers to the sustained psychological tension experienced by individuals during the employment preparation process due to a mismatch between external challenges and internal resources. For example, some researchers define employment pressure as the subjective perception and stress response of individuals to both internal and external stressors during the job-seeking process (Tu and Liu, 2025; Peng et al., 2024). Kwak et al. (2025) emphasize that university students experience anxiety during job-seeking due to a lack of social support and insufficient use of personal strengths, which negatively affects their mental health and quality of life. Yang et al. (2025) found that academic stress negatively impacts psychological resilience, thereby increasing employment anxiety, highlighting the important role of psychological resilience in alleviating employment pressure. Additionally, Wu et al. (2024), through latent profile analysis, discovered that the employment pressure of rural college students can be divided into two categories: high-level and low-level. The high-level group exhibited higher anxiety and lower self-efficacy in terms of employment psychology.

In summary, despite differences in expression, most viewpoints agree that the logic behind the generation of employment pressure aligns with the basic framework of interaction theory. This study adopts the definition by Tu and Liu (2025), viewing employment pressure as the subjective perception and stress response formed through the interaction of internal and external factors.

In this study, the term employment pressure is used to refer to the stress and tension experienced by university students during the processes of job search and career preparation. Consistent with the existing literature on employment-related stress, we conceptualize employment pressure as students’ overall appraisal of the difficulty of obtaining employment and the subjective burden associated with it.

#### 2.3.3 Measurement tools for employment pressure

Quantitative research on employment pressure primarily relies on questionnaire surveys. Various measurement tools have been developed specifically for Chinese university students, reflecting a diverse trend in measurement dimensions and content. For example, the “College Students’ employment pressure Source Questionnaire” developed by Shu and Tang (2007) covers six dimensions, including social environment, family relationships, and school employment guidance. The “College Students’ employment pressure Questionnaire” proposed by Chen and Jiang (2009) is more detailed in structure, setting six dimensions, including professional quality, employment competition environment, and employment psychological expectations, with a total of 59 items. Zhang et al. (2011) developed a questionnaire that includes five dimensions, such as employment support and career ambiguity, which has been tested for reliability and validity and is suitable for assessing employment pressure among university students. Based on interaction theory, Tang (2020) developed the “College Students’ employment pressure Scale,” which includes five dimensions and comprehensively assesses stressors and responses, demonstrating high reliability and validity. Chen and Zhao (2024) effectively assessed students’ employment psychological stress and related influencing factors by combining the GA-BP algorithm model with a mental health scale.

In this study, the College Students’ Employment Stress Scale developed by Chen and Jiang (2009) is used as the measurement instrument. This scale has been widely applied among Chinese university students. Its items focus specifically on the concrete difficulties and psychological experiences encountered by students in the job-seeking process, covering multiple aspects such as evaluation of vocational qualities, job-search competition, self-understanding and career positioning, and employment-related expectations. The scale adopts a Likert-type response format, with higher scores indicating higher levels of perceived employment pressure. Theoretically, the dimensions covered by this scale are highly consistent with the above conceptual definition of employment pressure and align with the transactional model’s understanding of the sources of stress (Chang and DeSimone, 2001). Therefore, the use of this instrument is both practically appropriate and theoretically consistent with the conceptual framework of the present study.

### 2.4 The interaction between peer relationships and employment pressure

In the context of higher education, peer relationships and employment pressure are often viewed as independent variables influencing students’ psychological states and academic performance. However, an increasing number of studies suggest that there may be a complex interaction between these two factors, collectively impacting university students’ learning wellbeing (Zhang et al., 2024).

On one hand, peer relationships, as an important social support resource, can alleviate the anxiety and psychological burdens caused by employment uncertainty through emotional companionship, academic assistance, and the construction of a sense of belonging. Positive peer interactions may enhance individuals’ confidence and adaptability towards the future, thereby reducing the negative effects of employment pressure on their learning motivation and wellbeing (Alsarrani et al., 2023). On the other hand, employment pressure itself may also have a feedback effect on the quality of peer interactions. When students face high levels of employment anxiety, they may rely more on peers for emotional guidance and practical information support, thus making peer relationships a mediator or buffering mechanism that moderates the effects of employment pressure (Mao and Zhao, 2023).

Based on this, the present study hypothesizes that peer relationships not only directly affect learning wellbeing but may also play a moderating role between employment pressure and wellbeing. In other words, under conditions of strong peer support, even when individuals experience high levels of employment pressure, their learning wellbeing may still be maintained at a relatively high level.

This study intends to further verify the above mechanism through interaction regression analysis and explore whether there is a statistically significant moderating effect between the two factors. The aim is to reveal the dual social and psychological mechanisms underlying the process of learning wellbeing among university students.

### 2.5 Research hypotheses

Based on the above theoretical analysis, the present study proposes the following hypotheses:

*H1*: Among post-2000 university students, interpersonal distress is significantly and negatively associated with learning wellbeing. Higher levels of interpersonal distress are expected to be related to lower levels of learning wellbeing across cognitive, emotional, quality-of-life, and growth dimensions.

*H2*: Employment pressure is significantly associated with learning wellbeing. In general, higher anticipated employment pressure is expected to be related to lower levels of learning wellbeing; however, a moderate level of employment pressure may show a certain positive association with cognitive and emotional dimensions of learning wellbeing.

H3: Interpersonal distress moderates the association between employment pressure and learning wellbeing. Specifically, among students with lower interpersonal distress, the negative association between employment pressure and learning wellbeing is expected to be weaker, whereas among students with higher interpersonal distress, the negative association between employment pressure and learning wellbeing is expected to be more pronounced.

## 3 Research methodology

This study employs a combination of questionnaire surveys and quantitative analysis to systematically evaluate the relationships among peer relationships, employment pressure, and learning wellbeing. The research methodology includes participant recruitment, selection of measurement tools, survey procedures, and statistical analysis. The specific details are as follows.

### 3.1 Participants

The participants in this study were full-time undergraduate students from Fujian University of Technology, Fujian Normal University, Sanming University, and Southwest Forestry University, all of whom belonged to the “post-2000” generation of university students. A stratified convenience sampling method was adopted: first, strata were established according to university, grade level, and major; then, within each stratum, several classes were selected based on accessibility, and all students in the selected classes were invited to complete the questionnaire. A total of 650 questionnaires were distributed, and 600 valid responses were obtained after data screening, yielding a valid response rate of 92.3%. Questionnaires were excluded as invalid if they met any of the following criteria: more than 10% missing data, completion time of less than 3 min, or over 80% of the items answered with the same response option.

The final sample ranged in age from 18 to 23 years, consistent with the age characteristics of the post-2000 cohort of university students. The detailed sample composition was as follows: in terms of grade level, 22.5% were first-year students, 24.5% were second-year students, 26.5% were third-year students, and 26.5% were fourth-year students. Regarding gender, 51.5% were male and 48.5% were female. With respect to academic discipline, 50.3% majored in humanities and social sciences, and 49.7% majored in science and engineering. Based on their most recent end-of-term examination results, academic performance was distributed as follows: 31.8% scored 90 and above, 40.7% scored 80–89, 16.3% scored 70–79, and 11.2% scored below 70. All participants took part in the study voluntarily and provided informed consent prior to completing the questionnaire.

### 3.2 Measures

This study employs three standardized questionnaires to measure learning wellbeing, peer relationship quality (interpersonal distress), and employment pressure levels. All scales have been validated in Chinese university student populations, demonstrating good reliability and validity.

#### 3.2.1 College students’ learning wellbeing questionnaire

The College Students’ Learning Well-Being Questionnaire, developed by Gao (2014), is used to measure learning wellbeing and includes two subscales: Subjective Learning Well-being and Psychological Learning Well-Being. It covers four dimensions: Cognitive Well-Being, Emotional Well-Being, Quality of Life Well-Being, and Growth Well-Being. The scale consists of 21 items and uses a 5-point Likert scale (1 = Strongly Disagree, 5 = Strongly Agree). In this study, the split-half reliability of the scale was 0.971, and the Cronbach’s *α* coefficient was 0.939, indicating good reliability.

#### 3.2.2 Interpersonal relationship comprehensive diagnostic scale

The Interpersonal Relationship Comprehensive Diagnostic Scale, developed by Zhang and Zheng (2004), is used to assess the degree of interpersonal distress, indirectly reflecting the quality of peer relationships. The scale consists of 28 items across four dimensions: Interpersonal Communication, Interpersonal Friendship, Social Interactions, and Heterosexual Relationships. Each item was scored dichotomously, with “no such problem” coded as 0 and “has such a problem” coded as 1. The item scores were summed to obtain a total score (theoretical range: 0–28), with higher total scores indicating more interpersonal problems, that is, higher interpersonal distress and lower peer relationship quality. To facilitate interpretation and comparison in the regression analyses, we further computed an interpersonal distress index based on the total score: the sum of the 28 item scores was divided by 28 to obtain an average score ranging from 0 to 1, representing the proportion of interpersonal distress items endorsed by the individual. Thus, the theoretical range of this index is 0–1, and higher values indicate more severe interpersonal distress and lower peer relationship quality. In all subsequent analyses, the “interpersonal distress” variable was described and modeled using this 0–1 index. In the present study, the split-half reliability of this scale was 0.971 and Cronbach’s *α* was 0.917, indicating good reliability.

#### 3.2.3 College students’ employment pressure questionnaire

The College Students’ Employment Pressure Questionnaire, developed by Chen and Jiang (2009), consists of 59 items and covers six dimensions: Professional Competence Evaluation, Job Seeking Competition, Self-awareness and Positioning, Employment Psychological Expectations, Lack of Job-seeking Assistance, and Supply–demand Imbalance in Majors. The scale uses a 5-point scale (1 = No Pressure, 5 = Very High Pressure). In this study, the split-half reliability of the scale was 0.989, and the Cronbach’s α coefficient was 0.986, with good reliability.

In the data used in this study, the Cronbach’s α coefficient and split-half reliability of the scale were both within an acceptable range, indicating good internal consistency reliability. It should be emphasized that these indices are used only to assess the reliability of the scale and do not constitute evidence of validity. Because no exploratory factor analysis (EFA) or confirmatory factor analysis (CFA) was conducted in the present study, the structural validity of the scale remains to be further examined in future research.

### 3.3 Procedure

The questionnaires in this study were administered using a combination of online and offline methods. For the offline component, course instructors or counselors distributed paper-and-pencil questionnaires during class or class meetings and collected them on site. For the online component, a questionnaire link was distributed via the university academic affairs system or class WeChat groups, and each student was allowed to submit the questionnaire only once using a unique account or student ID. All questionnaires were completed independently by the participants in a distraction-free environment. The researchers explained the purpose of the study, the principles of anonymity and confidentiality, and emphasized that participation was entirely voluntary and that participants could withdraw at any time.

During data consolidation, responses from the paper questionnaires were manually entered into an electronic spreadsheet and then merged with data exported from the online platform using a unified scheme for variable naming and coding. To prevent duplicate submissions, we screened for potential duplicates by comparing student IDs/class information, submission time, and highly similar response patterns, retaining only the earliest and most complete record. To ensure response quality, in addition to excluding questionnaires with a completion time of less than 3 min and those with more than 10% missing data, we also discarded questionnaires in which more than 80% of the items were answered with the same option. All questionnaires were accompanied by an informed consent form, and no personally identifiable information was collected.

### 3.4 Statistical analysis

SPSS software was used to analyze the data in this study. First, Cronbach’s *α* coefficients and split-half reliability coefficients were calculated for the three questionnaires to examine the internal consistency reliability of the scales. It should be noted that these indices are used to evaluate the reliability rather than the validity of the instruments. As no exploratory factor analysis (EFA) or confirmatory factor analysis (CFA) was conducted in the present study, the structural validity of the scales cannot yet be fully established and should be further examined in future research.

Second, descriptive statistical analyses were performed for the main variables and their dimensions, including interpersonal distress, employment pressure, and learning wellbeing. Means, standard deviations, and observed score ranges were calculated. Pearson correlation analyses were then conducted to examine the bivariate relationships among the variables and their subdimensions.

Third, to test the main effects and moderating effects proposed in the research hypotheses, multiple linear regression models were constructed. Before creating the interaction term, the continuous independent variables (interpersonal distress and employment pressure) were mean-centered to reduce the risk of multicollinearity. The interaction term was then computed by multiplying the centered interpersonal distress and employment pressure variables, and this term was entered into the regression model together with the main-effect variables. Variance inflation factors (VIFs) were calculated to assess whether multicollinearity was within an acceptable range, and residual plots were used to examine the assumptions of normality and homoscedasticity of the residuals. When the regression coefficient of the interaction term was statistically significant, simple slope analyses were further conducted to compare the predictive slopes of learning wellbeing under different levels of interpersonal distress and employment pressure, thereby clarifying the direction and strength of the moderating effect.

## 4 Research results

### 4.1 Descriptive statistics

Descriptive statistical analysis was conducted on the data from the 600 valid questionnaires, presenting the distribution characteristics of the variables related to peer relationships, interpersonal distress, employment pressure, and learning wellbeing. As shown in Table 1, the average score for learning wellbeing was 3.65, falling between “neutral” and “somewhat agree,” with a tendency towards “somewhat agree,” indicating that the overall sample exhibited a relatively high level of learning wellbeing. For the interpersonal distress variable, because the Interpersonal Relationship Comprehensive Diagnostic Scale consists of 28 yes/no items, each item in this study was scored as 0 (“no such problem”) or 1 (“has such a problem”), and the mean of the 28 items was computed to obtain an interpersonal distress index ranging from 0 to 1. This index can be understood as the proportion of interpersonal distress items endorsed by the participant; higher values indicate a greater number of reported interpersonal problems, that is, higher interpersonal distress and lower peer relationship quality. The average score for peer relationships was 0.66, suggesting that students experienced a certain degree of distress in their peer relationships. The average score for employment pressure was 3.50, indicating that most students perceived a moderate-to-high level of employment pressure.

**Table 1:** Descriptive statistics for peer relationships, employment pressure, and learning wellbeing.

Variable	Mean (M)	Standard deviation (SD)	Minimum (Min)	Maximum (Max)
Learning wellbeing	3.65	1.00	1	5
Peer relationships	0.66	0.48	0	1
Employment pressure	3.50	1.00	1	5

### 4.2 Correlation analysis

To further explore the linear relationships among variables, Pearson correlation analysis was conducted to examine the correlation coefficients between the four dimensions of learning wellbeing (cognitive wellbeing, emotional wellbeing, and growth wellbeing, quality of life wellbeing) and the four secondary dimensions of peer relationships (interpersonal communication, interpersonal friendship, social interactions, and heterosexual relationships), as well as the six secondary dimensions of employment pressure (professional competence pressure, job competition pressure, self-awareness and positioning pressure, employment psychological expectation pressure, lack of job-seeking assistance pressure, and supply–demand imbalance pressure). The results are presented in Table 2.

**Table 2:** Correlation matrix of peer relationships, employment pressure, and learning wellbeing subscales for post-2000 generation university students.

Learning wellbeing subscale	Learning wellbeing dimension	Professional competence pressure	Job competition pressure	Self-awareness and positioning pressure	Employment psychological expectation pressure	Lack of job-seeking assistance pressure	Supply–demand imbalance pressure	Interpersonal communication	Interpersonal friendship	Social interactions	Heterosexual relationships
Subjective learning wellbeing	Cognitive wellbeing	−0.520**	−0.510**	−0.490**	−0.500**	−0.480**	−0.530**	−0.450**	−0.420**	−0.430**	−0.440**
	Emotional wellbeing	0.400**	−0.390**	−0.370**	−0.380**	−0.360**	−0.410**	−0.300**	−0.290**	−0.310**	−0.320**
Psychological learning wellbeing	Growth wellbeing	−0.430**	−0.420**	−0.400**	−0.410**	−0.390**	−0.440**	−0.330**	−0.320**	−0.340**	−0.350**
	Quality of life wellbeing	−0.420**	−0.410**	−0.390**	−0.400**	−0.380**	−0.430**	−0.320**	−0.310**	−0.330**	−0.340**

Significance levels:*p < 0.05, **p < 0.01.

The analysis indicates that all dimensions of interpersonal distress (peer relationships) are significantly negatively correlated with the four dimensions of learning wellbeing (*p* < 0.01), suggesting that higher interpersonal distress is associated with lower learning wellbeing. Additionally, the six dimensions of employment pressure also show significant negative correlations with learning wellbeing, particularly “self-awareness and positioning pressure” and “employment psychological expectation pressure,” which exhibit higher correlation coefficients, indicating a stronger association with students’ wellbeing. Furthermore, there is a generally significant positive correlation between the dimensions of interpersonal distress and employment pressure, suggesting that higher levels of interpersonal problems are associated with higher perceived employment pressure.

### 4.3 Regression analysis: main effects and interaction effects

To examine the predictive effects of peer relationships and employment pressure on learning wellbeing, as well as their interaction effects, multiple regression models were constructed for each of the four dimensions of learning wellbeing (cognitive wellbeing, emotional wellbeing, quality of life wellbeing, and growth wellbeing) as dependent variables. Peer relationships (interpersonal distress), employment pressure, and the interaction term (peer relationships × employment pressure) were included as independent variables in each model. The results are presented in Table 3. The regression results are as follows:

**Table 3:** Regression analysis for peer relationships, employment pressure, and learning wellbeing for post-2000 generation university students.

Learning wellbeing dimension	Variable	Regression Coefficient (*β*)	Standard Error (SE)	*t*-value	Significance (*p*)	*R* ^2^
Cognitive wellbeing	Peer relationships	−0.251	0.060	−4.18	<0.01	0.105
	Employment pressure	0.150	0.070	2.14	<0.05	
	Peer relationships ×employment pressure	0.007	0.150	4.67	<0.01	
Emotional wellbeing	Peer relationships	−0.200	0.060	−3.56	<0.01	0.061
	Employment pressure	0.122	0.070	1.74	<0.05	
	Peer relationships × employment pressure	0.650	0.160	4.06	<0.01	
Growth wellbeing	Peer relationships	−0.156	0.040	−3.90	<0.01	0.023
	Employment pressure	−0.007	0.035	−0.20	>0.05	
	Peer relationships × employment pressure	0.500	0.150	3.33	<0.01	
Quality of life wellbeing	Peer relationships	−0.214	0.050	−4.78	<0.01	0.051
	Employment pressure	0.045	0.045	1.00	>0.05	
	Peer relationships × employment pressure	0.600	0.150	4.00	<0.001	

#### 4.3.1 Cognitive wellbeing regression analysis

**Figure 1:**
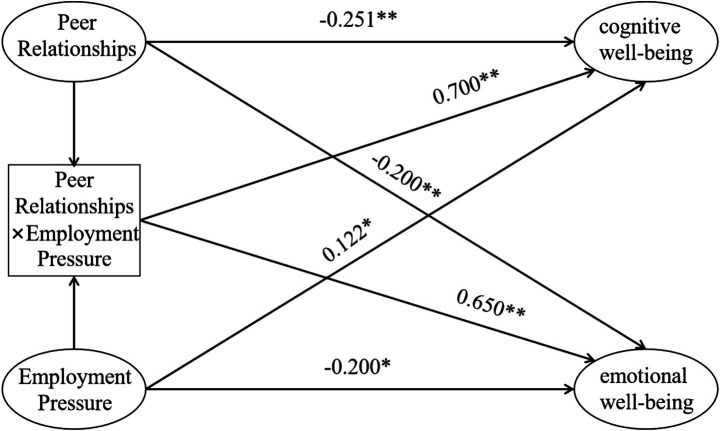
Pathway diagram of peer relationships, employment pressure, and the factors of subjective learning wellbeing.

In the regression model for cognitive wellbeing, interpersonal distress was a significant negative predictor (*β* = −0.251, *p* < 0.01), indicating that higher levels of interpersonal distress (i.e., lower peer relationship quality) were associated with lower cognitive wellbeing. Employment pressure had a significant positive effect on cognitive wellbeing (*β* = 0.150, *p* < 0.05), suggesting that, within the range observed in this sample, employment-related demands may be linked to a stronger sense of academic goal-setting and task engagement. The interaction term between interpersonal distress and employment pressure also significantly predicted cognitive wellbeing (*β* = 0.700, *p* < 0.01), indicating that the association between employment pressure and cognitive wellbeing varies as a function of students’ interpersonal distress levels. Simple slope analyses showed that this association was different for students reporting lower versus higher interpersonal distress (see Figure 1). However, given that the overall explanatory power of the model was modest (*R*^2^ = 0.105), the moderation effect should be interpreted with caution.

#### 4.3.2 Emotional wellbeing regression analysis

In the regression model for emotional wellbeing, interpersonal distress had a significant negative predictive effect (*β* = −0.200, *p* < 0.01), indicating that students who reported more interpersonal problems tended to experience lower emotional wellbeing in learning. Employment pressure showed a significant positive effect (*β* = 0.122, *p* < 0.05), suggesting that, to some extent, higher perceived employment demands may be related to greater emotional activation in the learning context. The regression coefficient for the interaction term between interpersonal distress and employment pressure was also significantly positive (*β* = 0.650, *p* < 0.01), indicating that the relationship between employment pressure and emotional wellbeing differs across levels of interpersonal distress. In other words, employment pressure is related to emotional wellbeing in different ways for students with lower versus higher interpersonal distress (see Figure 1). The explanatory power of the model was *R*^2^ = 0.061, again pointing to a modest effect size that should be interpreted carefully.

#### 4.3.3 Quality of life wellbeing regression analysis

**Figure 2:**
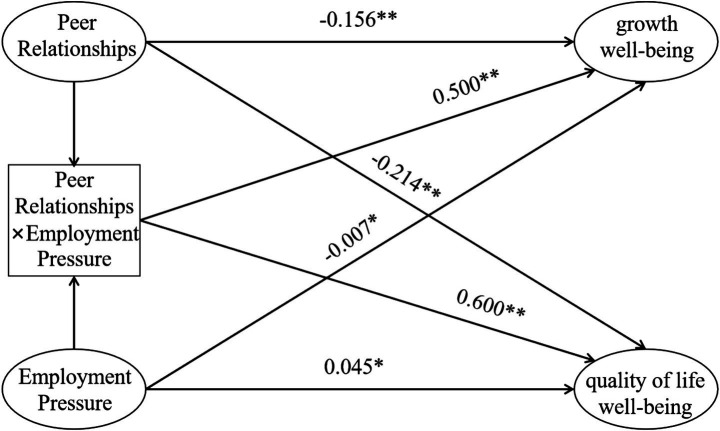
Pathway diagram of peer relationships, employment pressure, and the factors of psychological learning wellbeing.

In the regression model for quality of life wellbeing, interpersonal distress remained a significant negative predictor (*β* = −0.156, *p* < 0.01), meaning that higher levels of interpersonal conflict or loneliness were associated with a lower sense of quality of life in the academic domain. Employment pressure had no significant direct effect on quality of life wellbeing (*β* = −0.007, *p* > 0.05), suggesting that this dimension of wellbeing is less directly linked to perceived employment pressure. However, the interaction term between interpersonal distress and employment pressure was significant (*β* = 0.500, *p* < 0.01), indicating that the relationship between employment pressure and quality of life wellbeing changes depending on students’ levels of interpersonal distress (see Figure 2). Given that the overall explanatory power of the model was low (*R*^2^ = 0.023), this moderation effect should be regarded as small in magnitude and interpreted with caution.

#### 4.3.4 Growth wellbeing regression analysis

In the regression model for growth wellbeing, interpersonal distress continued to show a significant negative predictive effect (*β* = −0.214, *p* < 0.01), indicating that students who experienced more interpersonal difficulties reported lower levels of perceived growth in learning. The direct predictive effect of employment pressure was not significant (*β* = 0.045, *p* > 0.05), suggesting that employment pressure alone was not clearly related to growth wellbeing in this sample. The interaction term between interpersonal distress and employment pressure had a significant predictive effect (*β* = 0.600, *p* < 0.01), indicating that the association between employment pressure and growth wellbeing differs across levels of interpersonal distress (see Figure 2). However, the overall explanatory power of the model was modest (*R*^2^ = 0.051), and thus the practical significance of this moderation effect is limited. Rather than concluding that growth wellbeing “primarily depends on stable peer support,” it is more appropriate to state that interpersonal distress shows a small but detectable moderating role that warrants further investigation.

In summary, interpersonal distress was a significant negative predictor for all dimensions of learning wellbeing, whereas employment pressure showed a mixed pattern, with small positive associations in some dimensions and non-significant effects in others. Significant interaction terms were observed in all models, indicating that the associations between employment pressure and learning wellbeing differ depending on students’ levels of interpersonal distress. However, the additional variance explained by these interaction terms was small, so the moderation effects should be interpreted as modest and tentative rather than as strong psychological determinants.

## 5 Discussion

This study investigates the impact of peer relationships and employment pressure on learning wellbeing and their interaction mechanisms, using Post-2000s university students as the sample. Through regression analysis and interaction effect testing, the following key findings emerged: (1) Peer relationships have a significant positive predictive effect on learning wellbeing; (2) The impact of employment pressure on learning wellbeing shows a differentiated trend across different dimensions; (3) Peer relationships have a moderating effect on the relationship between employment pressure and learning wellbeing.

### 5.1 Direct effect of peer relationships on learning wellbeing

The results of this study show that interpersonal distress is significantly negatively correlated with all dimensions of learning wellbeing. Students who report more interpersonal problems tend to have lower scores in cognitive wellbeing, emotional wellbeing, quality-of-life wellbeing, and growth wellbeing. This finding is generally consistent with previous research on the associations between interpersonal difficulties, subjective wellbeing, and adaptive functioning among university students (e.g., Zhang et al., 2024), and it highlights the important role of positive interpersonal relationships in individual wellbeing. In the context of Chinese higher education, peers are not only academic collaborators but also important sources of emotional companionship and everyday support. Therefore, when students experience more conflict, isolation, or a lack of understanding in interpersonal interactions, their sense of belonging and perceived support may be weakened, which in turn can negatively affect their learning experiences and level of learning wellbeing.

### 5.2 The multidimensional impact of employment pressure

This study found that employment pressure does not have a purely negative effect. In the dimensions of cognitive wellbeing and emotional wellbeing, moderate employment pressure increased students’ awareness of learning goals and motivation. This finding partially supports the “stress-promoting theory” and the “positive stress model”(Peng et al., 2024). Moderate pressure may stimulate students to actively seek growth opportunities, fostering a sense of achievement and value in their learning process. However, it is also noteworthy that in the dimensions of quality of life wellbeing and growth wellbeing, the main effect of employment pressure was not significant. This suggests that excessive pressure may not directly translate into positive psychological experiences and may, in fact, lead to reverse effects due to a lack of supportive systems.

### 5.3 The moderating role of peer relationships

A particularly noteworthy finding is that interpersonal distress plays a moderating role in the association between employment pressure and learning wellbeing. Across several dimensions of learning wellbeing, the interaction between interpersonal distress and employment pressure reached statistical significance, indicating that the impact of employment pressure is not uniform for all students but varies as a function of interpersonal distress. This conclusion is largely consistent with the results of existing literature (Worley et al., 2023). Specifically, the simple slope analyses suggested that, among students with higher interpersonal distress (i.e., poorer peer relationship quality), the association between employment pressure and learning wellbeing tended to be less favorable (weaker positive associations or stronger negative associations). In contrast, among students with lower interpersonal distress (relatively better peer relationship quality), the association between employment pressure and learning wellbeing was relatively more favorable.

These findings suggest that interpersonal distress may have a risk-amplifying function: when students experience both high employment pressure and high interpersonal distress, they are more likely to report reduced learning wellbeing. Conversely, when interpersonal distress is low, the potential adverse association between employment pressure and learning wellbeing appears to be attenuated. However, the explanatory power of the models constructed in this study was relatively low (*R*^2^ ranged from 0.023 to 0.105), indicating that although the interaction effects reached statistical significance, their effect sizes were modest and the related conclusions should be interpreted with caution. Future research could incorporate additional variables such as psychological resilience and learning motivation to build more comprehensive theoretical models.

### 5.4 Theoretical contributions and practical implications

From a theoretical perspective, this study integrates social relationship variables and stress variables to explore their joint effects on learning wellbeing, expanding our understanding of the mechanisms that form students’ psychological wellbeing. On a practical level, the findings suggest that universities should focus on fostering a positive peer interaction atmosphere, organizing group counseling sessions and peer support programs within classes to buffer the negative effects of employment pressure. Furthermore, moderately increasing students’ awareness of employment challenges will help transform stress into a source of growth.

### 5.5 Limitations and future directions

Despite the empirical findings of this study, there are several limitations: First, the data collection mainly relied on self-reported questionnaires, which may be influenced by subjective bias and social expectations; second, the study sample was concentrated in Fujian and southwestern regions, limiting the representativeness of the findings; third, this study employed a cross-sectional design, which does not allow for the exploration of causal relationships between variables.

Future research could consider employing a longitudinal tracking design, combining behavioral measurements and physiological indicators, to further explore the dynamic roles of peer interaction quality and employment pressure in long-term development. Additionally, incorporating mediating variables (such as psychological resilience and academic motivation) could help construct a more comprehensive theoretical model.

## 6 Conclusion

This study investigates the impact of peer relationships and employment pressure on university students’ learning wellbeing, using a quantitative questionnaire survey method to analyze data from 600 valid samples of Post-2000s university students. The study systematically explores the main effects and interaction relationships among these three factors, leading to the following key conclusions:

First, interpersonal distress is a significant negative predictor of learning wellbeing. Students who experience more interpersonal problems tend to report lower learning wellbeing across multiple dimensions, including cognitive, emotional, quality-of-life, and growth aspects. This highlights the importance of healthy peer interactions in building students’ psychological wellbeing.

Third, interpersonal distress plays a moderating role in the relationship between employment pressure and learning wellbeing. The results suggest that higher interpersonal distress is associated with a less favorable pattern of learning wellbeing under employment pressure, whereas lower interpersonal distress (relatively better peer relationship quality) is associated with a more favorable pattern. When students face both high employment pressure and high interpersonal distress, their wellbeing is more likely to decline, indicating a potential “double risk” profile. However, the additional variance explained by the interaction terms is small, so this moderating effect should be viewed as modest, and the present findings should not be overinterpreted as strong evidence of a powerful buffering effect.

In summary, the formation of university students’ learning wellbeing is a multidimensional, multifactorial process of interaction. This study not only expands the theoretical boundaries of research on learning wellbeing but also provides practical intervention suggestions for universities in areas such as student support, psychological development, and career guidance.

## Data Availability

The original contributions presented in the study are included in the article/supplementary material, further inquiries can be directed to the corresponding author.
